# To take charge of one's life - group-based education for patients with type 2 diabetes in primary care - a lifeworld approach

**DOI:** 10.1080/17482631.2020.1726856

**Published:** 2020-02-11

**Authors:** Anna Kjellsdotter, Mia Berglund, Elisabeth Jebens, Jennie Kvick, Susanne Andersson

**Affiliations:** a Skaraborg Hospital Skövde, Research and Development Centre, Skövde, Sweden; b School of Health Sciences, Skövde University, Skövde, Sweden; c Primary Health Care Center, Stenstorp, Sweden; d Primary Health Care Center Mösseberg, Falköping, Sweden; e Department of Health Sciences, University West, Trollhättan, Sweden

**Keywords:** Diabetes nurse, group-based education, learning, lifeworld, phenomenology, type 2 diabetes

## Abstract

**Background**: The number of people suffering from diabetes worldwide, including Sweden, has increased. To strengthen the patient’s empowerment and thus improve their ability to take care of their own health, patient education in self-care management plays a central role in diabetes care.

**Purpose**: The specific aim in this study was to describe patients’ experiences of group-based education using the *Taking charge of one’s life with type 2 diabetes* model.

**Methods**: A qualitative approach with a phenomenological lifeworld perspective was used. The study was based on group and individual interviews and reflection books.

**Results**: The group-based education model made it possible for the patients to learn through reflection concerning their own and others’ experiences. The learning that occurred with support from the group reflections and the reflection books contributed to the understanding of the complexity of the illness. This increased the motivation and desire to be responsible for the treatment and implementation of habits. The group contributed to a sense of belonging and community that inspired a continued and active learning.

**Conclusion**: The results showed that from the patients’ perspective, this didactic model was both suitable and appreciated, supporting and facilitating learning.

## Introduction

Diabetes is one of the most common, long-term illnesses in the western world entailing significant changes to one’s life. The number of people suffering from diabetes worldwide has increased from 108 million in 1980 to 422 million in 2014 (World Health Organisation, 2018). The prevalence of known type 2 diabetes (T2D) in Sweden today is estimated to be in the order of 4–5%. In addition, 10–15% have a precursor, prediabetes, with a high risk of developing T2D. Reduced physical activity and increased occurrence of obesity in the population are important contributing factors. The prevalence of T2D increases with age and is estimated at 10–20% in people over 65 years of age. A connection has been found between unsatisfactory blood glucose control and the occurrence of diabetic-late complications (NDR, 2015). Structured group-based patient education gives a significant reduction of HbA1c (SBU, 2009; Scain, Friedman, & Gross, 2009). Therefore, patient education in self-care management supported by a diabetes nurse plays a central role in diabetes care in order to strengthen the patient’s empowerment and thus improve their ability to take care of their own health (Jutterström, Hörnsten, Sandström, Stenlund, & Isaksson, 2016; The National Board of Health and Welfare, 2015).

Self-care in T2D often requires a variety of changes in living habits such as dietary changes, increased exercise, smoking cessation, medical treatment and constant blood glucose monitoring. It has been found that the ability to grasp the illness goes hand in hand with the ability to handle one’s health. When a certain turning point is reached and the patient realizes that they are suffering from a lifelong illness, a better balance in managing health is achieved not only on an existential and emotional level, but also on a practical level (Hörnsten, Jutterström, Audulv, & Lundman, 2011).

Learning to live with diabetes can be understood as a struggle between objectifying and incorporating the illness. By finding a balance in this struggle, the illness may become a new part of the person’s life, creating a new way of life (Johansson, Österberg, Leksell, & Berglund, 2018; Kneck, Klang, & Fagerberg, 2012). In order to balance life, the patient needs to learn to feel and reflect on what is important for well-being. Questions and thoughts that lead to reflection are an important part of the learning process. Patients often imagine a dark and scary picture of the future with a long-term illness and avoid talking about it. However, it has been found that by talking about their anxiety and acquiring knowledge of the opportunities to create a good future despite the illness, the ability to deal with their anxiety is facilitated (Berglund, 2014). A long-term illness affects all aspects of life and is therefore difficult to ignore in everyday life. Furthermore, learning affects the whole person, the body, cognitive functions, emotions, practical issues and social life. This complexity must be understood for genuine learning to be possible. The learning process can be understood as a movement to a new understanding that is shown by the way the person acts and lives his/her life with the illness (Berglund & Källerwald, 2012). Increased self-awareness facilitates more thoughtful choices. In addition, it is important for the learning process to set goals; these goals are often to maintain independence and focus on what gives meaning to life (Berglund, 2014).

This research on patients’ learning is based on the lifeworld theory. From the lifeworld perspective, the human body is understood as a lived body that is, at the same time, existential and biological—thinking, feeling and acting (Merleau-Ponty, 2002/1945). The concept of lifeworld refers to the theories of intentionality and the natural attitude. According to these theories’ consciousness is directed towards something other than oneself (Husserl, 1907/1989). In the natural attitude learning takes place unconsciously, for example when we do things. But learning can also through reflection take place on a more conscious level. Learning from the lifeworld perspective differs from other theories of learning as focus is on the whole (thinking, feeling and acting) instead of one specific aspect, for example behaviour. From a lifeworld perspective learning implies a changed understanding of things in the world, and of who I am i.e., a learning on an existential level. Learning shows itself in how we act and is supported through reflection and dialogues that involve the whole being in the person’s context (Bengtsson, 2006; Berglund, 2014; Ekebergh, 2007). Learning is an individual process, but it is supported in a community with others. Learning to live with long-term illness occurs when the person reflects on experiences and how events are related to wellness (Berglund, 2011).

Learning can be supported by considering the patient’s life context and the understanding of their situation (Berglund, 2011). In order to support the patient in the learning process, the healthcare professional must have insight into how the illness affects the patient’s everyday lives and an understanding of how the illness affects the perception of context and meaningfulness (Dahlberg & Segesten, 2010). The healthcare professional’s support has to be based on the patient’s learning needs and not solely on a fully-structured care programme (Newman, Steed, & Mulligan, 2004). Further, it is important for the learning process to combine the theoretical knowledge with a person’s own actions in order to understand the context and to make the knowledge concrete and related (Miettinen, 2000). Learning to live with diabetes is about letting the unusual become everyday again. This means that the new situation with the disease or illness is integrated and becomes natural in a person’s life situation (Johansson, Österberg, Leksell, & Berglund, 2015).

Social interaction is important in education and helps adults to absorb information and improve their sense of belonging, as well as offering an opportunity to build a support network (New, 2010). A group-based educational program based on a social constructivist model of learning for newly diagnosed patients with T2D can lead to improved quality of life and self-care behaviour. By promoting active learning that is related to personal experience, the education gives a sense of being in control of diabetes management (O’Brien, Cardwell, Nair, & Hardy, 2015). Group-based self-management education is associated with fewer acute complications and is rated as more “useful” by patients than individual counselling. Patients value being able to share problems with similarly affected people (Hwee, Cauch-Dudek, Victor, Ng, & Shah, 2014). When patient education takes place on a horizontal basis, the general knowledge of the healthcare professional and the unique knowledge of the patients are valued equally. The educator’s task is to be a supervisor and to provide group members with the time and tools for self-reflection and the exchange of experience; the patient’s ability to participate in their care and to take responsibility for their health is strengthened (Adolfsson, Starrin, Smide, & Wikblad, 2008; Andersson, Svanström, Ek, Rosen, & Berglund, 2015). There are many obstacles to improving self-management that can be related to a lack of person-centred care and health literacy. Information from the education program can change knowledge, but it often has very little impact on behaviour (Laursen, Frølich, & Christensen, 2017). It is the diabetes nurse’s task to offer the most appropriate form of education for each person. Therefore, it is important to seek knowledge about how group-based education supports patients with T2D in their own learning and how the teaching should be designed to meet the needs of the patients in the best possible way. The overall objective was to implement and evaluate the didactic model (Berglund, 2011) entitled *Take charge of one’s life—learning by long-term illness* in group-based education of people with T2D in primary care, both from a patient perspective and from a healthcare professional perspective. The healthcare professional perspective was described in an earlier study (Andersson, Berglund, Vestman, & Kjellsdotter, 2019). The specific aim in this study was to describe patients’ experiences of group-based education using the *Taking charge of one’s life with T2D* model.

### Taking charge of one’s life with T2D

The group-based education model has its origin in the lifeworld didactic model: The challenge—to take charge in life with a long‐term illness (Berglund, 2011). The model aims to support patients’ learning on an existential level (Table I).

**Table I.** Four principles of the didactic model. A tactful challenging approach is profound in the model

The challenge to take charge of life with a long-term illness	
1	Confronting one’s life situation and challenging oneself to make a changes
2	Positioning oneself at a distance while creating a new whole
3	Developing self-consciousness and taking responsibility, shifting from ‘one’ to ‘I’
4	Making learning visible with the aim of achieving development and balance in life

The didactic model is based on a dialogue with patients using a tactful, challenging, didactic approach based on the patient’s life situation, problems and issues. During the dialogue, the diabetes nurse asks questions that support the reflection and help the patient verbalize his/her own situation, what it is like and possible ways to handle it. The intention is that the patient should feel his/her own power and see and use this to take charge of his/her own life situation and more directly express goals for the treatment and life in general (Berglund, 2014). This model has been tested and studied in the context of haemodialysis (Andersson et al., 2015). In this study, the four theses in the didactic model (Table I) have been converted to five themes (Table II) adapted for group-based education *Taking charge of one’s life with T2D*. Each group session has its own theme.

**Table II.** The themes reflected upon during the group sessions

Session 1	My idea of the illness T2D
Session 2	Who am I? - Who am I with T2D?
Session 3	Past - Present – Future
Session 4	Obstacles - Opportunities, Strengths - Weaknesses
Session 5	My goals, Challenges - My own learning process

The healthcare professional’s role is to create a reflective dialogue with the patients in addition to the participants’ questions about illness, treatment and life in relation to the themes. The healthcare professionals who facilitate the education sessions had graduated from a university course about the didactic model based on a lifeworld approach. A previous study from a professional perspective showed that the specially trained personnel experienced that group education made it possible for patients to learn through reflection, concerning both their own and others’ experiences. Further, the healthcare professionals expressed that they learned a lot from the patients (Andersson et al., 2019). As a supplement to the themes and group sessions, the patients had to write reflection notes on themselves in a reflective book. Written and verbal reflections were made based on the following: the past, present and future, strengths and difficulties, and short and long-term goals. In addition, the participants discussed their reflections in the group.

## Method

In this qualitative study, the phenomenon—patients’ experience of group-based education—from the model *Taking charge of one’s life with T2D* was explored and illuminated using the reflective lifeworld research (RLR) approach, based on phenomenological epistemology (Dahlberg, Dahlberg, & Nyström, 2008).

### Setting and participants

The patients who participated in this study were recruited at two primary healthcare centres in western Sweden. Twelve patients with T2D, five women (mean age 71.0 years) and seven men (mean age 70.6 years) participated in the study. Verbal and written informed consent were obtained from participants. Inclusion criteria were that the diagnosis should not be older than three years and not less than three months. Exclusion criteria were a history of cerebrovascular disease or another severe disease, known drug abuse and/or difficulties understanding or writing the Swedish language that made it impossible to participate in group-based education. No selection was made regarding age or gender.

### Procedures

The education consisted of five group sessions (Table II) and comprised approximately two hours per session. During each education session, the focus was on different topics and each session started with a presentation of the theme of the day. This was then followed by a round of presentations of the patients’ questions and thoughts from the previous session. The rounds were repeated during the session so that everyone was given the opportunity to speak. At the end of each session, the patients were given the opportunity to reflect on their experiences and their learning in a personal reflection book. Reflections on the past, present and the future were elicited.

### Data collection

Before the group-based education started, two group interviews were carried out at baseline. After completion of the education, individual telephone interviews were conducted. Interviews, both individual and group interviews, included a questionnaire with open questions. Group interviews conducted at baseline focused on expectations and concerns. The initial question was: Can you tell us about your expectations concerning group-based education? The introductory question captured their experience of group education. In the follow-up questions, it was important to be open and listen to the answers and let the group members discuss and reflect on what was said. In this way, a broader and deeper perspective was created of the experiences that existed in the group and which were reflected on. The group interviews lasted 33 and 40 minutes, respectively. After completion of the didactic education, the focus moved to their experiences concerning the participation in group-based education. The individual telephone interviews were initiated with the question: Can you tell me about your group-based education experience? In-depth questions were asked: Can you tell me more? Can you give examples? How do you mean? These twelve interviews lasted between 15 and 38 minutes.

After each education session, patients were invited to reflect on their experiences and their learning in a reflection book. These reflections were collected for analysis after approval by the participant. The reflection books each comprised 30 pages.

### Data analysis

The interviews were audiotaped and transcribed verbatim. The data comprised 160 single space text pages. All data were read repeatedly in an open manner by the researchers, both individually and together, to create understanding and a thorough knowledge of the text. Using reflective lifeworld research (RLR), a research approach developed by Dahlberg et al. (2008) was used in order to describe the phenomena and their meaning. The analysis was directed towards discovering patterns and nuances of qualitative meanings that emerged from the transcribed text. The analysis was characterized by openness and sensitiveness in an intensive dialogue with the text. This dialogue vacillated from parts to whole with the aim of deepening the understanding of the phenomenon. All data were divided into meaning units, a sequence of the text that has a meaning of its own. Each meaning was reflected against the background of the whole. The next phase involved building groups of meanings, as clusters. Finally, the essence was formed, which is described by Dahlberg (2006) as an abstraction and synthesis of a phenomenon’s unique structure of meanings. The essence with structure and nuances of the phenomenon can be understood as a new whole. The reflection books were read and processed in the same way as the transcribed interviews. In this paper, the essence is presented in the results, followed by the five constituent elements of the phenomenon, illustrated with quotations from the interviews.

### Ethical considerations

The Regional Ethical Review Board approved the study (Dnr: 442–15). Informed and written consent was obtained from all participants in the study. Principles according to the Helsinki declaration were followed (WMA, 2008).

## Results

The essence of the phenomenon—participants’ experiences of group-based education from the *“Taking charge of one’s life with T2D”* model involves individual learning and changes, the person changes their understanding of the disease and it becomes a part of their life. The learning takes place through raised issues and reflections concerning experiences on a concrete and existential level which clarifies responsibility and motivates changes. The group contribute to a sense of togetherness and community that inspires continued and active learning. The increased understanding and self-awareness motivate changes in living habits and achievements of own goals. The reflections in the group together with own experiences and reflections that occur between the meetings support the fact that the disease is incorporated with the person’s self-image and life, which constitute learning on an existential level. Learning was supported by a tactful challenging approach. The phenomenon being studied was further enlightened by its five constituents: learning from each other, increased self-awareness, finding motivation to change, clarifying your own responsibility and learning through experiences.

### Learning from each other

Learning from each other meant sharing experiences by listening to others’ experiences and challenging each other to change; this process was motivated by reflection, curiosity, and a desire to understand. Group-based education gave them the opportunity to contribute with their own experiences, thereby contributing to the learning of others. In this way, an exchange of experiences took place that gave them an experience of gaining a lot of knowledge, but also gaining someone to share their concerns with. In turn, this gave a feeling of not being alone in the situation. Participants claimed that in individual counselling, there was a greater risk that questions fell into oblivion and were therefore never asked.

It was very rewarding and lots of fun … lots of questions that I maybe wouldn’t have thought of popped up … other people asked the sort of questions that you might have thought of yourself …

The participants stated that knowledge obtained in lecture form often provided basic theoretical knowledge, it was not described as being as worthwhile as learning from each other. Group learning required basic knowledge about the disease and nutrition to be able to reflect and ask specific questions. Reflection on the risk of disease complications and their implications on health led to existential issues. Knowledge that the risk of complications increases depending on the onset of the illness, gave an insight into the importance of making the necessary changes in their living habits.

About risks in the future … well it is obvious that this was completely new to me. I thought it was interesting … [the discussion in the group] … and then I checked on the Internet and studied it a bit.

Then I have come to realize that … it really affects me.

The importance of learning from each other also appeared in the different ways of reflecting. The opportunity to raise their problems in the group and even write them down gave time to reflect on their own life situation and the consequences of the illness. Reflecting together in a group and sharing each other’s thoughts and opinions were experienced as easier than reflecting individually and putting thoughts in writing. Individual reflections and writing down their own thoughts and experiences in the reflection books were experienced as a bit more difficult and required more time and instruction. By following up individual reflections in the group, participants were encouraged to do “homework”.

Because I am not the sort of person who can suddenly sit at a table with six other people round me and suddenly write down something there. No, my mind was blocked. I thought it was really difficult … well, I wrote a bit at home and … well I had to think about what … well, I thought it was hard.

Learning from each other was expressed in the social aspects of the phenomenon. Group sessions contributed to a feeling of increased community and an opportunity to make new acquaintances. Opportunity was given for interactive discussions with a lot of laughter, but also the chance to raise difficult questions for mutual reflection in a tactful challenge to take charge of life with a long-term illness. There were variations in how open and outgoing everybody in the group was. For those who did not find it easy to talk about themselves to others, there was still some pleasure expressed in listening actively and sharing the stories and experiences of others.

The discussions that arose in the group often revealed the same or similar problems, thoughts and concerns; it was a good forum where experiences could be exchanged, and opportunities were made available to share their own knowledge with others. Cohesion in the group was reinforced by the feeling of sharing concerns and the knowledge of not being alone. It also gave a sense of togetherness described as an interest in knowing what happened to the other participants.

But it would be nice to meet in a year and see what happened … there were six or seven … what has happened to us? What has this person done and what has that person not done …

### Increased self-awareness

Diabetes is an illness that the patient can, to a great extent, influence and this becomes evident in the participants’ learning process. Experience of the group-based education using the *Taking charge of one’s life with T2D* model increased self-awareness. The realization of one’s own situation that was shared with others in the group gave them not only self-insight, but also an increased understanding that living with diabetes was different for everyone. The participant’s described how they realized that the illness influenced their life situation and challenged them to make changes. It also raised awareness that the illness may have adverse health consequences unless it was taken seriously, and that they took responsibility for their health.

I decide about my life, not the diabetes. It is obvious that my illness influences my life in both a positive and negative way. I have diabetes. I have to live with it. It all depends on whether my blood sugar is high or low. Me and nobody else rules over my life for better or worse.

Although some of the participants thought they already lived a healthy life with a healthy diet and exercise and could not understand why they had been affected by diabetes, it became obvious that there were still small changes that could be made and that they could make more conscious choices. It became obvious that changes were necessary; the insight that the illness is complicated also became equally clear. Furthermore, a new understanding related to the severity of the illness was described. The risks of diabetes with complications were a new insight for some of the participants and that really affected them. During group-based education, it became obvious that conditions were different for everyone. It appeared that there was some prejudice about the disease that needed to be re-evaluated.

It was good to find out what others had done and what problems other people had but then I still didn’t understand … there was a bloke … he was about my age, and he was as thin as a rake, but he had huge problems with the blood sugar levels, and then I think … what’s he doing wrong or what … he had huge problems as far as I could understand … to get his levels down.

The participants described that they actually had some knowledge about how they should live with a proper diet and exercise, but motivation was lacking. Still, they realized how important it was to take the illness seriously and make wise choices to live as good a life as possible without complications.

### Finding motivation to change

Fear of consequential diseases was a key driver when integrating the illness into one’s life and motivated changes in living habits that are necessary because of T2D. Motivation was not self-evident and was described as a deficiency that may hinder the success of the changes expected of those suffering from the illness. The participants describe that they had become self-aware by confronting the reality and complexity of the illness in the group. This was especially evident when the participants saw the needs and the resistance to change, which existed for other people in the group. The participants meant that with support of the conversation in the group and with help of reflective tools such as pictures and drawings, the awareness increased. Furthermore, there was anxiety about not knowing the consequences of diabetes. It was experienced as difficult to get insight about being affected by a serious and lifelong illness. A diagnosis that requires more or less major changes in life can be difficult to understand when the symptoms are few or nonexistent. If the participants’ health was not significantly affected and complications felt very far away, this influenced motivation to change. However, with the support of group reflections on “the past, present and future” (a tool in the reflective book); guilt and shame about suffering from T2D, and the fears for the future including what the disease may lead to later in life were processed.

Me who is overweight and fat … well that isn’t good, is it … I have never been ill with diabetes or well … now I have never been ill at any time so that I have noticed anything. But it is this, what will happen tomorrow or the day after or what …

Something that increased the motivation was to meet other people and learn together in a group. Listening to and sharing each other’s experiences inspired and increased motivation to address their own situation. This self-insight was affected by the others, such as seeing each other’s successes and adversities increased their motivation and inspiration to make changes that may be significant for increased well-being and lead to improved results of blood sugar levels.

### Clarifying your own responsibility

It became clear during the group-based education that it was up to everyone to take responsibility for their own health and treatment. The illness was described to be constantly present and something that they must always keep in mind. This can be thought of as a limitation of life with emotions such as anger, sadness, frustration and feelings of compulsion. These feelings were manifested in such a way that the perception of freedom was lost. Freedom did not entail the same as before, i.e., being able to eat what was offered; now they must apologize and refrain. In addition, exercise was described as something previously experienced as fun, but nowadays it felt like something forced because it was essential to control the disease. Consequently, some of the pleasure was lost.

I do it because I must … get exercise and the like. Yes, I must. It’s all about going outside even if it’s freezing, yes and it feels like I’m forced to. But before I did it voluntarily, now it feels more like I must go out, now you’re thinking about the sugar.

Although the experience of the illness was manifested in sacrifices and negative feelings such as frustration, anger, sadness and compulsion, it became obvious that they are themselves responsible and no one else could take responsibility for their treatment. It also appeared that the openness and support provided from the healthcare professional’s challenges encouraged them to reflect about their own goals, expectations and needs. By being challenged in the group, the participants realized that they had to take charge, instead of the illness taking control of them.

### Learning through experiences

Through the group-based education using the *Taking charge of one’s life with T2D* model, new knowledge was transformed into new and changed habits. The learning took place when theoretical knowledge about T2D, its complications and how it affects the body were reflected in relation to their life situation. Reflections concerning experiences made the participants make conscious choices. Furthermore, information on what affects blood glucose levels was the basis for understanding the relationship between diet, exercise and life habits. When it became apparent that blood glucose levels, blood pressure and weight improve, this was confirmation that knowledge had been acquired.

I’m waiting for the long-term sugar to be able to see if things are going in the right direction all the time … because if they are, then I think … then you get a little reward for what you’re doing. Then it’s all worth it.

During group-based education, it became clear that a concrete way of learning was through personal experience. A separate blood glucose measuring device could be used to see the actual impact of the diet and the effects of physical exercise. This device increased knowledge and was a practical tool that could be used in everyday life. The positive experience of learning through experience was described by a participant in the group: “we tried various recipes … checked our blood sugar before and then 1½ hours later. Yes, well then you sort of know what is good and what isn’t good to eat.” Even the use of a pedometer as part of learning clarified the image of yourself and your living habits. This was expressed by a lady in the group who was surprised because “I think that I am never still … was so very still when I counted at the end of the day.” The pedometer gave an experience of instantly seeing the link between cause and effect. It was very clear that by measuring blood glucose, based on the new knowledge, and choosing better dietary alternatives resulted in better blood glucose levels in the long term. With the newly acquired knowledge, it became more difficult to ignore the possible consequences of the disease. Making learning visible through reflections concerning experiences also entailed personal development and balance in life.

## Discussion

The results showed that this model of group-based education supported reflective learning by transforming experiences to new knowledge and skills by thinking, doing and feeling. The patients stated that it was very rewarding to learn from each other and make use of each other’s experiences. In addition to what was conveyed by a diabetes nurse and dietician, the results showed that the group provided a unique opportunity to glean information from other patients’ experiences of living with the illness. The group members motivated and supported each other in changing their life habits while providing each other with social support. The experience of this model of group-based education activated reflection, increased self-awareness and supported the patient’s ability to handle the treatment. Furthermore, other studies support the fact that group-based education with people with T2D result in improvements in living habits, and clinical and psychosocial outcomes (Johansson, Österberg, Leksell, & Berglund, 2016; Steinsbekk, Rygg, Lisulo, Rise, & Fretheim, 2012). Knowledge gained by a diabetes nurse and dietician provided valuable information, but the knowledge of living with diabetes mainly came when the patients shared their own experiences with each other. The result of learning from each other may be transferred to other patient groups in need of support and where changes in living habits contribute to increased health and insight into the nature of the disease. The present study showed that although the purpose of group-based education was essentially to learn and acquire knowledge, it transpired that the social aspects were very important to the participants. Some patients lived alone and did not have a large social network. For these people, it was particularly valuable to get new contacts.

Learning through reflections concerning experiences, which can be understood as an experiential learning, is very rewarding. According to Miettinen (2000), this gives an opportunity to see the direct link between cause and effect. Tests using a blood glucose measuring device and pedometers led to knowledge and understanding of how diet and exercise affected the measured values. Furthermore, a review of research in the field of teaching self-management with chronic illness revealed that nurse-led teaching and support over time combined with self-directed experiential learning, where participants can try themselves, led to improved knowledge and skills (Pinchera, Dellolacono, & Lawles, 2018). Results of other studies confirm that technology can be used to supplement diabetes care, with positive impacts on glycaemic control, self-management behaviours, and self-efficacy (Durán et al., 2010; Johansson et al., 2016). With this in mind and together with reflection over experiences, blood glucose measurement devices and pedometers should be used more generously for educational purposes to strengthen the patients’ own learning.

This model of group-based education provided the participants not only with theoretical knowledge that the illness was progressive, but it also provided an awareness through reflection that each person was responsible for his or her own health. According to other research, this emerges when the patient integrates the illness emotionally and existentially, learning through reflection and taking responsibility for understanding their own body (Johansson et al., 2015; Jutterström et al., 2016). The reflection supported by the model of group-based education led to increased insight into the illness and that it was mainly the patient’s responsibility to take care of their own health and treatment. This took a lot of time and, for some, it was a burden to try to change their living habits. When it became clear that diet and exercise were an important part of the treatment of the disease, it became more of a compulsion for some to get exercise and read the contents on food packaging, while others saw it as an opportunity to improve their values and hopefully avoid later complications of the disease. In terms of preventive care, lifestyle changes were the focus. The ethical aspects of patient education were highlighted by Berglund (2011) who discusses whether nurses have the right to force new knowledge on patients; this is knowledge that patients currently do not consider necessary. Therefore, the patients need support to realize that if the illness has not become an integral part of everyday life, it may interfere and take an even larger part of life later on, i.e., with increased risk of complications.

In order to be able to work more with empowerment and group-based education, a new approach is needed where it is not the patient’s outcome that becomes governing; rather, it is the patient’s ability to integrate the illness into their everyday life with T2D that is paramount. In order to support the patient’s ability to take responsibility for their own health, the goal needs to be more person-centred and based on the patient’s lifeworld (Johansson et al., 2018). The didactic model used in this group-based education, as opposed to traditional group education, aimed to go into more depth in terms of how the illness changes the patient as a person, and thus, to a greater extent, how he or she can control life with T2D. This was accomplished by giving the patients the opportunity to reflect both in groups and individually in their reflection books. The reflections contained thoughts, feelings and actions, thus leading the patient to make more informed choices, consequently managing their own care and health in a way that they decided by themselves, i.e., not based on goals set by caregivers. The model required the nurses to learn to listen with an open mind, curiously and lecture the patient less. The nurse’s most important tasks will then be to lead the reflection, and to deepen it in a way that involves the participants on both an emotional and concrete practical level. This is in line with other research that argue for that healthcare professionals need to learn to accept and respect the patient’s choices, as they do not always align with what is considered most important to the patient (Anderson & Funnell, 2010). Patients with diabetes become more active and show greater satisfaction with the care when it is based on their individual needs (Boström, Isaksson, Lundman, Lehuluante, & Hörnsten, 2014; Jutterström et al., 2016). Patients usually strive to be healthy and prevent complications of their illness. Healthcare professionals may know what is best for the patient’s diabetes disease, but that does not mean that particular knowledge is best for the individual patient’s life. Everyone has their own unique lifeworld, which a healthcare professional is not always privy to. It is important to be patient and respect the patient’s set goals instead of always focusing on the care goals. Healthcare professionals must put trust in the fact that the patient is striving to achieve the best possible health, based on their individual conditions. In order to achieve good care, healthcare professionals need to be more responsive to the patient and his/her needs, instead of expecting the patient to be responsive to external care goals.

A strength of this study was the fact that interviews were conducted both in groups and at an individual level complemented by reflection books (Dahlberg, 2014). The written text was a description of lived experiences and provided a strong connection to the lifeworld. Accessing the reflection books that the participants wrote during the group-based education gave clearer and more thoughtful answers than the interviews. This meant that those patients who were apprehensive about speaking in front of the group still had the opportunity to express their thoughts and opinions. The reflection books also gave more time for reflection and probably raised questions that could be addressed at future meetings. Having access to group interviews, individual interviews and reflection books provided a more varied holistic set of data. During the analysis, it became clear that learning through issues and reflections concerning experiences on a concrete and existential level was the core of the phenomenon together with learning from each other; together these were essential throughout the material and emerged as the essence of the phenomenon. The lifeworld theory is part of the didactic model as well as a foundation for the research (RLR). This can be seen as a strength in the study, but it could also have been seen as a weakness if the lifeworld perspective had not included the approach of openness and critical reflection of the understanding of the phenomenon in the research process. Dahlberg and Dahlberg (2003) describe this as being bridled in relation to the phenomenon studied.

## Conclusion

Group-based education based on the *Taking charge of one’s life with T2D from the patients’ perspective* model is a feasible and appreciated form of education that supports and facilitates learning through reflection and dialogue. Reflecting together with other people who have diabetes and sharing experiences contributes to the group’s own learning. Based on this, it can be concluded that learning to live with diabetes based on the patient’s lifeworld perspective supports a more effective and pragmatic learning environment.

## Practice implication and future research

The primary application of this approach is to support the learning process for those living with a lifelong illness such as diabetes. This study highlights the importance of practical applications of models drawn from research. The didactic model can be useful in making care more person-centered and in achieving learning that challenges patients to take charge of their own care.
